# Somatosensory Amplification, Anxiety, and Depression in Patients With Hepatitis B: Impact on Functionality: Erratum

**DOI:** 10.1097/01.md.0000489592.74957.b2

**Published:** 2016-07-18

In the article “Somatosensory Amplification, Anxiety, and Depression in Patients With Hepatitis B: Impact on Functionality”,^1^ which appeared in Volume 95, Issue 21 of *Medicine*, the *P* values in Table 2 were incorrectly given. The article has since been corrected online.
